# The Differential Diagnosis of Primary Iritis in the Early Stages

**Published:** 1931

**Authors:** E. R. Chambers

**Affiliations:** Ophthalmic Surgeon, Bristol Royal Infirmary; Surgeon, Bristol Eye Hospital


### THE DIFFERENTIAL DIAGNOSIS OF PRIMARY IRITIS IN THE EARLY STAGES.

BY

E. R. Chambers, F.R.C.S.,

*Ophthalmic Surgeon, Bristol Royal Infirmary;*
*Surgeon, Bristol Eye Hospital.*

---

Primary iritis is an inflammation which begins in the iris itself, and is not the result of some other morbid process in the eye. Except, perhaps, in the traumatic variety, the condition is caused by a blood-borne infection. An eye so attacked manifests certain cardinal signs and symptoms which must be carefully looked for and their significance appreciated.

A. *Circumcorneal injection.*—In the early stages this manifests itself as a reddish or lilac-coloured blush around the margin of the cornea and extending for some distance into the sclera. If this is carefully looked at with the naked eye or, better still, with any lens of low magnification, the vessels are seen to be straight and hair-like, radiating out from the corneal margin and situated beneath the conjunctiva.

B. *Changes in the iris itself.*—These manifest themselves in a change in the normal colour, loss of lustre and indistinctness of its pattern. These changes are all due to an inflammatory œdema of the iris itself, an exudation on the surface of the iris and perhaps a clouding of the aqueous with inflammatory products. Thus a blue iris becomes greenish and a brown iris yellow. The delicate crypts and folds become filled up, giving the iris a flat surface. The condition of the iris itself is, therefore, a very valuable guide, and if the opposite eye is unaffected a comparison should always be made of the irides in the two eyes.

C. *Contraction and impaired mobility of the pupil* result in the early stages from hyperæmia and spasm of the sphincter muscle.

A careful study of the symptoms is no less important.

1. *Pain* is often severe, and if carefully questioned the patient will say that it is not so much in the eye itself but rather in the brow, or the side of the nose, or that the whole side of the head is painful, and that it is neuralgic in character. From a differential diagnostic point of view it is very important to realize that iritis produces this type of pain.

2. *Lachrymation and photophobia* are due to reflex irritation of the fifth nerve. The fact that only tears are in excess and that there is no inflammatory discharge should be appreciated.

3. Diminution of vision is the result of the aforementioned conditions.

The common conditions from which an iritis has to be distinguished are:—

(*a*) Conjunctivitis.

(*b*) Episcleritis.

(*c*) Subacute glaucoma.

In *conjunctivitis* the eye is red, but if the hyperæmia be carefully investigated it will be seen that instead of the fine, straight, hair-like and lilac-coloured vessels situated around the circumference of the cornea, the vessels take their origin from arterial loops in the fornices of the conjunctiva, that their calibre is largest at a point farthest removed from the cornea, that they are brick-red, tortuous, and move with the conjunctiva. This is a very different picture from that seen in an early case of iritis. Then, again, as there is an inflammation of a surface membrane, there will be a definite discharge instead of the lachrymation of iritis. This discharge may be so slight that none is visible, but the patient will probably state that the lids are glued together on waking from sleep, owing to an accumulation of the discharge during the night. In conjunctivitis the iris itself has a normal appearance with a normal pupil, and no diminution in vision unless the discharge is sufficiently great to produce a mechanical obstacle to sight.

The pain in conjunctivitis is usually quite different from that of iritis, it consists of a gritty feeling, the patient often seeking advice for the removal of a foreign body from the eye. This is in contrast with the severe neuralgic pain of iritis, which is in the bones of the skull rather than in the eye itself. In the later stages of an iritis both the circumcorneal and conjunctival systems of blood-vessels become affected, but if care is taken to examine the iris itself the diagnosis will not be missed.

*Episcleritis* was a relatively rare disease a few years ago, but has recently become much more common. It occurs more frequently in the autumn months, and may assume epidemic proportions. It attacks adults of middle age, and is caused by an inflammation of the connective tissue between the conjunctiva and sclera, or the superficial layers of the sclera itself. Although the disease may show itself anywhere in the anterior part of the eye, the commonest situation is close to the corneal margin. The importance from a differential diagnostic point of view is that the inflammation, as a rule, attacks only a segment of the sclera, the rest of the eye being clear. Both the conjunctival and deeper vessels are involved, and the hyperæmia is often more purplish than red. In the more severe cases there is a definitely raised nodule in the centre of the inflamed patch. There is no discharge from the eye, but pain, lachrymation and photophobia. The pain is variable, sometimes hardly complained of, but in other cases it is severe, often of a neuralgic type, and situated in the bones of the orbit and side of face. The iris, at any rate in the early stages of the disease, is normal, the pupillary reactions also being unaffected. This condition is commonly mistaken for iritis, but the fact that the disease is localized to a segment of the sclera and that the iris is normal should present no difficulties of diagnosis.

I must also refer to that type of *subacute glaucoma* which presents only slight inflammatory signs, and is often the first appearance of the disease in an otherwise healthy eye. It may, on the other hand, occur during the progress of an already established chronic glaucoma of which the patient has made no complaint, the disease, therefore, not having been diagnosed. It is characterized by pain which the patient describes as aching in the eye itself, and perhaps more so in the bones of the orbit and side of the face. It is characteristic of the pain that it may have been severe in the early hours of the morning, that it wakened the patient from sleep, that it lasted perhaps an hour or so, but was considerably improved by the time he got up. The tendency is for such attacks to clear up completely, leaving the eye apparently normal, but further attacks are bound to occur until perhaps an acute bout is developed. If seen during the morning after one of these attacks, the eye may show a faint hyperæmia due to the enlargement of the circum-corneal blood-vessels, and may easily be confused with a very early attack of iritis, but the iris itself is normal in colour and pattern, the pupil may or may not show some tendency to dilatation, but is never contracted as in early iritis, and the anterior chamber is frequently of less depth than in a normal eye. In most cases, if the cornea be examined with the naked eye, it will have a normal appearance, but if any weak magnifying lens is used a slight clouding or perhaps a ground-glass appearance may be seen. This is due to an œdema of the corneal epithelium produced by the increase of the intra-ocular tension at the time of the attack, and may persist for some time. The diagnosis of glaucoma has, in the past, largely depended on the presence or not of an increase in the intra-ocular tension; but it is now known that at any rate in the early stages of the disease this may only be present for a few hours during the twenty-four, and although one should never fail to take the tension, its absence does not necessarily exclude the presence of glaucoma. In these early and transient attacks of glaucoma the fundus and optic discs are perfectly normal, pathological changes being present only if the subacute attack is grafted on a chronic condition.

I have endeavoured to emphasize the importance of these transient attacks of glaucoma, as little mention is made of them in the literature, and yet the importance of bearing them in mind cannot be over-estimated; for should an eye in this condition be mistaken for an early iritis, and atropine be used in the treatment, an acute glaucoma would inevitably result.

The differential diagnosis of iritis in the very earliest stage is of the utmost importance, for if it is recognized and adequately treated at once the duration of the disease is considerably lessened and vision often but little impaired; but its treatment necessitates the use of atropine, and before this potent drug is used there should be no possible doubt as to the diagnosis.

